# Molecular dynamics simulation and experimental study of electrochemical dissolution properties of 440C stainless steel

**DOI:** 10.1016/j.isci.2025.113845

**Published:** 2025-10-23

**Authors:** Xinyu Wen, Yuanlong Chen, Fankai Zhu, Xiang Li, Hua Lin, Wei Jiang

**Affiliations:** 1 School of Mechanical Engineering, Hefei University of Technology, No. 193, Tunxi Road, Hefei 230009, Anhui Province, China; 2 School of Intelligent Manufacturing, Anhui University of Applied Technology, No. 2600, Wenzhong Road, Hefei 230011, Anhui Province, China; 3 Anhui Province Key Laboratory of Critical Friction Pair for Advanced Equipment, Hefei 230088, Anhui Province, China; 4 School of Mechanical and Automotive Engineering, West Anhui University, Moon Island, Lu’an, Anhui Province, China

**Keywords:** electrochemistry, corrosion, electrochemical materials science, simulation in materials science

## Abstract

This study investigated the influence of electrolyte composition on the electrochemical dissolution behavior of 440C stainless steel. Dissolution characteristics were evaluated in six distinct electrolytes using electrochemical workstation. Molecular dynamics simulations were used to model electrolyte ion penetration through the passivation layer of 440C stainless steel during electrochemical corrosion. Results demonstrate that the 10% NaCl + 10% NaNO_3_ electrolyte leads to the greatest corrosion depth and dissolution rate. Experimental evaluation of the material removal rate (MRR) and post-dissolution surface roughness revealed that, although the 10% NaCl + 10% NaNO_3_ electrolyte achieves a higher MRR, it also causes greater surface roughness compared to other electrolytes. Furthermore, the application of external magnetic field during electrochemical dissolution was examined. The results indicate that the magnetic field enhances MRR, reduces surface roughness, improves dissolution efficiency, and ultimately improves the surface quality of the machined 440C stainless steel.

## Highlights

• A molecular dynamics model for simulating electrochemical corrosion
• Revealing electrolyte effects on corrosion of 440C stainless steel
• Demonstrating the magnetic field effect on 400C stainless steel corrosion

## Graphical abstract

**Figure:**
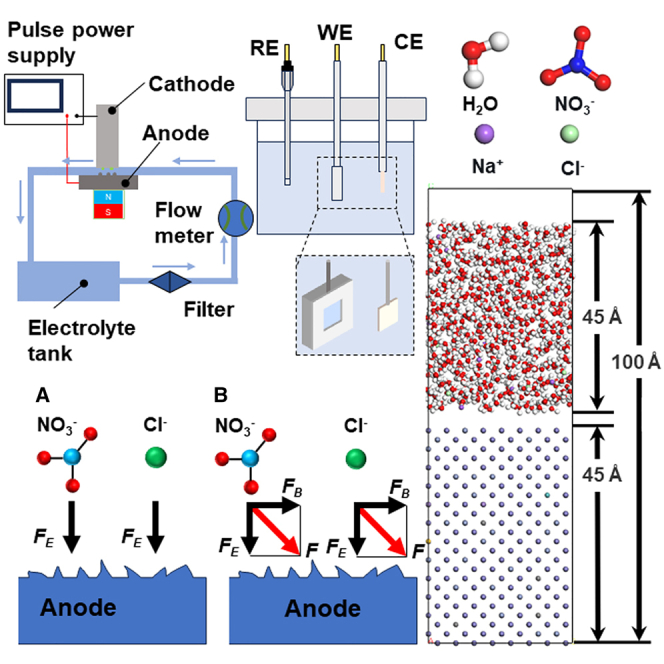

## Introduction

440C stainless steel, a high-carbon martensitic alloy acknowledged as a superhard material, is renowned for its excellent wear and corrosion resistance. It is commonly used as a material for cutting tools and bearings, but its high hardness poses significant challenges for conventional mechanical methods. Electrochemical machining (ECM) is a non-contact processing technique^1^^,^^2^^,^^3^ that avoids inducing thermal stress on the machined surface. Because this method is not constrained by the hardness or geometry of the workpiece, it is particularly well suited for the machining of superhard materials.^4^^,^^5^^,^^6^ In current ECM of stainless steel, neutral electrolytes are widely utilized due to their low corrosiveness, safety, reliability, and cost-effectiveness.^7^^,^^8^

Stainless steel exhibits excellent passivation behavior in neutral electrolytes such as NaNO_3_. The passive film formed on the surface acts as a physical barrier, significantly impeding metal ion dissolution and the cathodic reactions of reducing agents, thereby reducing the corrosion rate. This passivation also mitigates stray corrosion during ECM, improving overall machining quality.^9^ Numerous scholars have explored the effects of neutral electrolytes on the ECM of stainless steel. The impact of different cathode materials in ECM was studied using a mixed electrolyte of NaNO_3_ and C_6_H_8_O_7_.^10^ The results indicated that the oxide film of aluminum alloy 6061 effectively reduced stray corrosion, identifying it as a promising cathode material for the ECM of 304 stainless steel. The influence of magnetic field assistance during the ECM of 2Cr13 stainless steel in a NaNO_3_ solution was examined.^11^ It was found that increasing the magnetic field intensity reduced the surface roughness, with a value of 0.28 μm achieved under a magnetic flux density of 300 mT and a processing time of 14 min. A superhydrophobic pit array structure was fabricated on 304 stainless steel using ECM in NaNO_3_ solution, with pits spaced 300 μm apart and 98 μm deep. Micro-grooves and nanoparticle structures with excellent superhydrophobic and anti-reflective properties were formed at the pit edges.^12^ ECM of pores on the surface of 304 stainless steel was conducted in NaNO_3_ solution.^13^ Key parameters, including voltage, duty cycle, and cathode feed rate, were optimized using orthogonal methods. Evaluation criteria included hole taper, overcut rate, and material removal rate (MRR) to determine the optimal parameter combination for hole drilling quality. Annular micro-grooves were fabricated inside 304 stainless steel tubes via pulse ECM in NaCl electrolyte.^14^ A rotating tooth-shaped cathode was designed to optimize the flow field structure, resulting in grooves with a depth of 340 μm and a width of 263 μm.

During ECM, complex mass transfer occurs at the interface between the electrolyte and the anode. Traditional experimental methods are insufficient for analyzing ion transport and material removal mechanisms at the molecular level. Molecular dynamics (MD) simulations, which use Newtonian mechanics to model atomic motion, offer significant advantages in elucidating the mechanisms of action and reducing experimental cycles when they are used to study the changes at the molecular scale in materials during macroscopic machining. The formation of carbide on γ-Fe surfaces at 1,000–1,600 K was studied using MD simulations.^15^ Analysis of the atomic mechanisms involved an examination of the adsorption behavior of C and H atoms during the process at different temperatures. It was observed that high temperatures promoted the diffusion of C and H elements into the substrate, resulting in H desorption. The effect of particle size on the melting point of iron nanomaterials was investigated.^16^ It was found that the melting point of dual-particle systems was between the melting points of the individual particles, approaching the higher value as the size decreased. A coupled MD model for drilling was established to investigate dislocation behavior at speeds of 30, 60, and 90 m/s.^17^ The behavior was revealed to affect load stability.

Currently, the application of MD methods in conventional machining has garnered significant attention, with research demonstrating their effectiveness in modeling machining processes. However, its application in electrochemical corrosion remains relatively limited. Notably, electrochemical corrosion is a material removal process at the ionic level, making it well suited for investigation using MD. Exploring this application can elucidate reaction mechanisms at the atomic scale, offering valuable insights for future research. Therefore, this study used an electrochemical workstation to investigate the dissolution behavior of the superhard material 440C stainless steel in various neutral electrolyte. MD simulations were used to model the 440C stainless steel matrix and electrolytes, simulating the atomic-scale material removal process during electrochemical corrosion. An experimental setup was also established to electrochemically process 440C stainless steel in various electrolytes and under different magnetic field conditions. The machined surfaces were then characterized to examine the effects of electrolyte composition, concentration, and magnetic field on its electrochemical corrosion behavior.

## Results

**Figure 1:**
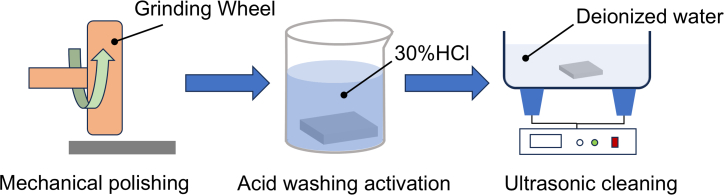
Substrate pre-treatment

The composition of the electrolyte significantly influences charge transfer and mass transport rates during electrochemical reactions. To investigate the dissolution behavior of 440C stainless steel in various neutral electrolytes, solutions with varying compositions and concentrations were prepared, as shown in Table 1. Each solute was fully dissolved, and the volume was adjusted to 1,000 mL to ensure consistent concentration. Prior to corrosion testing, the substrate underwent pre-treatment as shown in Figure 1.

**Table 1:** Electrolyte solution composition

Number	Solution	Composition	
		NaCl/g·L^−1^	NaNO_3_/g·L^−1^
A	5% NaCl	50	0
B	10% NaCl	100	0
C	5% NaNO_3_	0	50
D	10% NaNO_3_	0	100
E	5% NaCl + 5% NaNO_3_	50	50
F	10% NaCl + 10% NaNO_3_	100	100

**Figure 2:**
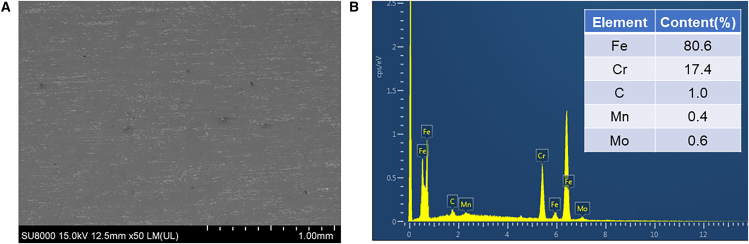
Surface morphology and elemental composition of 440C stainless steel (A) SEM plot; (B) EDS plot. Test samples are randomly selected from all samples.

The surface morphology and elemental composition of 440C stainless steel were characterized using scanning electron microscopy (CEM3000A, Chotest Technology Inc [chotest], China) and energy-dispersive spectroscopy (EDS). The results are shown in Figure 2. As illustrated in Figure 2A, mechanical polishing produced a relatively smooth surface, and EDS spectrum in Figure 2B presents the elemental composition of the 440C stainless steel.

**Figure 3:**
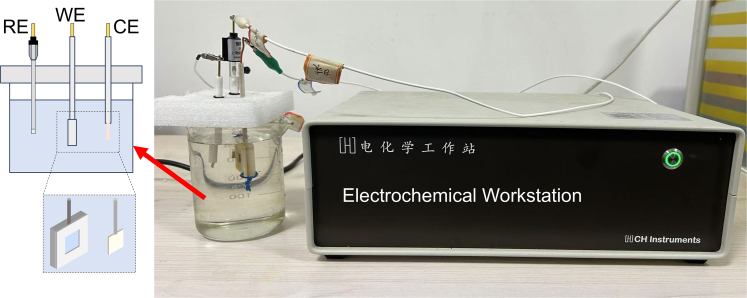
Diagram of the electrochemical testing process

The electrochemical testing procedures are shown in Figure 3, with the electrolyte temperature maintained at 25°C.

**Figure 4:**
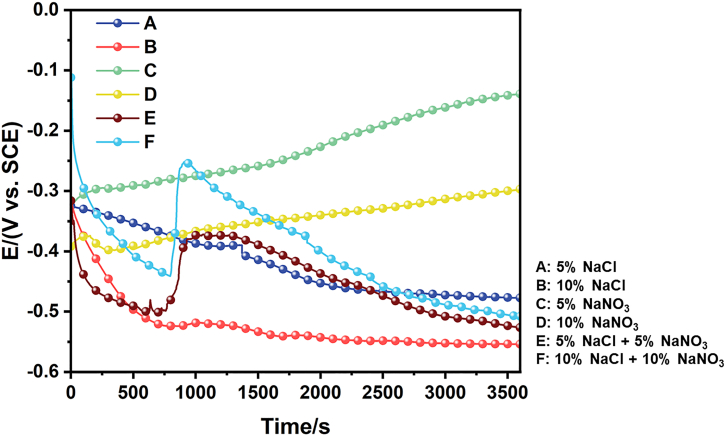
Open circuit potential-time curves of 440C stainless steel in various electrolytes

Figure 4 presents the open circuit potential (OCP) curves of 440C stainless steel in six distinct electrolytes. Significant variations in potential evolution are observed depending on the electrolyte composition and concentration. In NaCl solutions, the OCP shifts toward more negative values with increasing immersion time, with the 10% NaCl solution exhibiting the most negative potential (approximately −0.55 V vs. saturated calomel electrode). This indicates that chloride ions aggressively disrupt the passive film and promote active dissolution, with a higher chloride concentration leading to increased corrosion susceptibility of the alloy. Conversely, in NaNO_3_ solutions, the OCP gradually shifts in the positive direction and stabilizes after a certain immersion period. This behavior suggests that nitrate ions facilitate the formation and maintenance of a protective passive film on the 440C stainless steel surface. For mixed electrolytes, the OCP evolution demonstrates a competitive effect between chloride-induced film breakdown and nitrate-induced passivation. In the low-concentration mixed solution (E), the potential stabilizes around −0.5 V, reflecting a partial equilibrium between corrosion activation and passivation. In contrast, OCP in the high-concentration mixed solution (F) exhibits more pronounced fluctuations: partial recovery after an initial negative shift, ultimately stabilizing gradually. This unstable trend suggests that the passive film undergoes repeated breakdown and repair under the combined influence of aggressive chloride ions and protective nitrate ions.

In summary, the OCP results indicate that chloride ions shift the potential negatively due to their corrosive nature, while nitrate ions shift the potential positively by promoting passivation. In mixed electrolytes, the competition between these two processes determines the electrochemical stability of 440C stainless steel.

**Figure 5:**
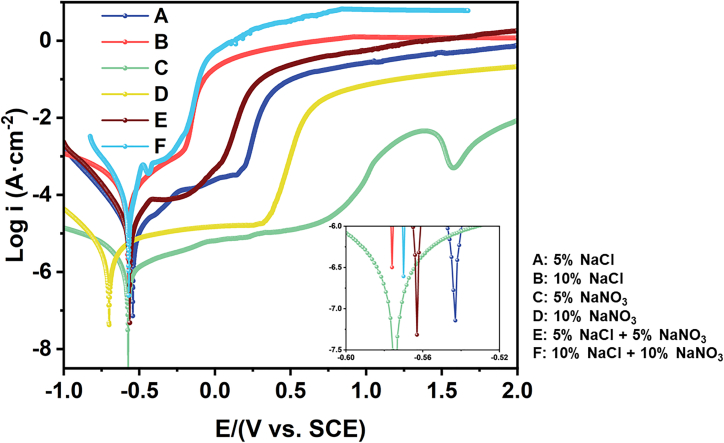
Polarization curves of 440C stainless steel in different electrolytes

The polarization behavior of 440C stainless steel in different electrolytes was investigated using the potentiodynamic scanning method over a potential range from −1 to 3 V. The resulting polarization curves, shown in Figure 5, reveal distinct passivation and over-passivation regions, as well as the breakdown potential of the passivation films formed in various electrolytes. Passivation current density (*i*_1_), variations in the passive potential (*E*_1_), transpassive potential (*E*_2_), and passive region width (Δ*E*_12_) in different electrolytes are summarized in Table 2. In NaNO_3_ electrolyte, the lower passivation potential (*E*_1_) indicates that the material more readily undergoes passivation, leading to the formation of a surface passive film. The higher transpassive potential (*E*_2_) suggests that NO_3_^−^ oxidation promotes the development of a chromium-enriched layer, which enhances resistance to high-valence-state dissolution. The passive range width (Δ*E*_12_) reflects the stability of the passive film; a larger Δ*E*_12_ indicates a more stable and protective passive layer on the surface.

**Table 2:** Electrochemical parameters of 440C stainless steel in different electrolytes

Electrolyte	Passivation current density *i*_1_/(μA·cm^−2^)	Passivation potential *E*_1_/mV	Over-passivation potential *E*_2_/mV	Range of passivation zones Δ*E*_12_/mV
A: 5% NaCl	74.9	−236	144	380
B: 10% NaCl	245.5	−472	−234	238
C: 5% NaNO_3_	1.8	−424	756	1,180
D: 10% NaNO_3_	5.5	−571	286	857
E: 5% NaCl + 5% NaNO_3_	124.7	−429	−74	355
F: 10% NaCl + 10% NaNO_3_	462.2	−458	−262	196

As observed in Figure 5, distinct passivation zones are evident. Within this region, the current density exhibits a gradual increase, indicating the formation of a passive film on the stainless steel surface, which provides corrosion resistance. However, as the potential further increases, a sharp rise in current density is observed, signifying the breakdown of the passive film and the onset of rapid material dissolution.^18^^,^^19^ Prior research indicates that a relatively extended passivation window suggests enhanced passivation capabilities of the electrolyte. The passivation range of a material in an electrolyte is directly proportional to the stability of the passive film, indicating higher corrosion resistance.^20^^,^^21^^,^^22^

According to Table 2, the passivation region of the material in electrolyte B (10% NaCl) is significantly smaller than that in electrolyte A (5% NaCl), and the passivation current density is higher. This suggests that the material is more susceptible to electrochemical corrosion and exhibits reduced passivation ability in high-concentration electrolytes. Similarly, the passivation region of the material in electrolyte D (10% NaNO_3_) is smaller than that in electrolyte C (5% NaNO_3_), with a higher passivation current density. Compared to NaCl electrolytes, the material exhibits a larger passivation region and a lower passivation current density in NaNO3 electrolytes, regardless of the concentration (5% or 10%). This indicates that the material passivates more readily in NaNO_3_ electrolytes, forming a dense passive film that inhibits further corrosion. The synergistic effect of Cl^−^ and NO_3_^−^ on the corrosion of the material can be observed from electrolytes B (10% NaCl), D (10% NaNO_3_), and E (5% NaCl +5% NaNO_3_). Table 2 shows that the passivation region and passivation current density of the material in electrolyte E (5% NaCl +5% NaNO_3_) are intermediate between those in electrolytes B (10% NaCl) and D (10% NaNO_3_). This suggests that the passivation effect of NO_3_^−^ mitigates the corrosion caused by Cl^−^ to some extent, leading to an increased passivation region and a decreased passivation current density of the material.

An in-depth analysis of the electrochemical reactions of 440C stainless steel in an electrolyte solution was conducted. During the corrosion process, oxidation occurs at the anode and reduction occurs at the cathode, with electron transfer as follows.

Cathode reaction:

(Equation 1) $$\left\{ \begin{array}{l} 2H_{2}O+2e^{-}\to H_{2}↑+2OH^{-}\\ 2H^{+}+2e^{-}\to H_{2}↑ \end{array}\right.$$

Anode reaction:

(Equation 2) $$\left\{ \begin{array}{l} M-ne^{-}\to M^{n+}\\ M^{n+}+nOH^{-}\to M{\left(OH\right)}_{n} \end{array}\right.$$

Subsequently, the hydrolysis of M(OH)_n_ yields various oxides, including Fe_2_O_3_, Fe_3_O_4_, and Cr_2_O_3_, etc.^23^

(Equation 3) $$nM{\left(OH\right)}_{n}\to M_{m}O_{\frac{nm}{2}}+\frac{nm}{2}H_{2}O$$

In summary, the passivation range of 440C stainless steel is the narrowest in the composite electrolyte containing 10% NaCl and 10% NaNO_3_, indicating that the passive film formed under this condition exhibits the poorest stability. Conversely, the widest passivation range is observed in the 5% NaNO_3_ solution, accompanied by a lower passivation current density. This suggests that the passive film formed in this electrolyte is the most stable and provides the highest corrosion resistance.

**Figure 6:**
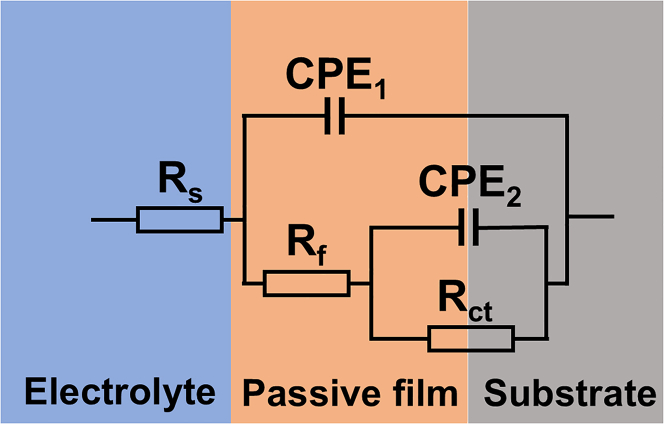
Equivalent circuit for corrosion of 440C stainless steel in different electrolytes

The equivalent circuit model of the passivation film on the surface of 440C stainless steel, fitted using ZView software, is shown in Figure 6.^24^^,^^25^^,^^26^

In this model, R_s_ represents the electrolyte resistance, R_f_ denotes resistance of the passivation film, CPE_1_ (Constant Phase Element) corresponds to the capacitance of the passivation film, R_ct_ indicates the charge transfer resistance at the film-substrate interface, and CPE_2_ accounts for the double-layer capacitance. The CPE is used to describe the non-ideal capacitive behavior of the passivation film, with its impedance defined as follows^27^:

(Equation 4) $$Z_{CPE}={\left[Q{\left(i\omega \right)}^{n}\right]}^{-1}\left(-1\leq n\leq 1\right)$$

where, *Q* is the CPE constant, *i* is the imaginary unit (*i*^2^ = −1), *ω* is the angular frequency (rad·s^−1^), and *n* is the CPE exponent. The behavior of the CPE depends on the value of *n*: capacitance (*n* = 1), Warburg impedance (*n* = 1), or resistance (*n* = 0).

**Figure 7:**
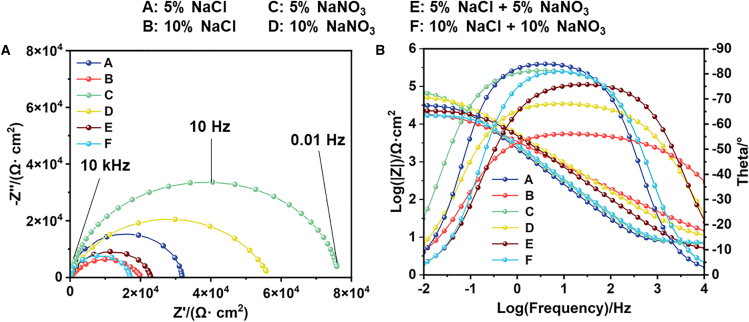
Impedance spectroscopy of 440C stainless steel in different electrolytes (A) Nyquist plot; (B) Bode plot.

The Nyquist and Bode plots of the impedance spectra for 440C stainless steel in different electrolytes are shown in Figure 7. According to electrochemical dissolution theory, a larger capacitive arc in the impedance spectrum indicates stronger corrosion resistance. As seen in Figure 7A, under the same electrolyte conditions, the impedance of 440C stainless steel is higher in low-concentration electrolytes, lower in the 10% NaCl + 10% NaNO_3_ electrolyte, and highest in the 5% NaNO_3_ electrolyte. In Figure 7B, the impedance amplitude at low frequencies corresponds to the impedance of the passivation film on the surface of 440C stainless steel, while the high-frequency amplitude reflects the electrolyte impedance. It is evident from Figure 7B that the impedance of 440C stainless steel in the 5% NaNO_3_ solution is the highest at low frequencies, with a phase angle close to 90°, indicating that the passivation film exhibits significant capacitive behavior. In Figure 7B, within the high-frequency domain, the impedance spectra of 440C stainless steel in NaNO_3_ solution exhibit a slope approaching −1.0, indicating the formation of a compact passive film and nearly ideal capacitive behavior at the interface. In the presence of NaCl, Cl^−^ compromises the passive film, leading to charge transfer resistance (R_t_) dominance and diminished capacitive behavior. The impedance spectra in 10% NaCl + 10% NaNO_3_ solution display the largest slope, reflecting significant non-ideal capacitive behavior, which is attributed to the aggressive impact of high concentrations of corrosive ions on the passive film surface.

Table 3 presents the equivalent circuit fitting data of the impedance spectra in different electrolytes. The goodness of fit was evaluated by chi-square test (χ^2^ test), with an order of magnitude of 10^−4^, indicating that the equivalent circuit model accurately represents the interface system of the passive film. The equivalent circuit used for fitting comprises two time constants: the first time constant (R_f_-CPE_1_) represents the passive film properties, while the second time constant (R_ct_-CPE_2_) corresponds to the charge transfer process occurring at the interface between the substrate and the film. Therefore, the distribution of time constants reveals the presence of two distinct but coupled electrochemical phenomena. Here, R_s_, R_f_, and R_ct_ represent the resistance of the electrolyte solution, the outer film, and the inner film, respectively. These time constants reflect the interplay between thin film formation, ion transport, and charge transfer at the metal surface.

**Table 3:** Equivalent circuit fitting data for 440C stainless steel in electrolytic solution

Electrolyte	R_s_ (Ω · cm^2^)	CPE_1_ × 10^−4^ (S · cm^−2^ · s^n^)	n_1_	R_f_ (Ω· cm^2^)	CPE_2_ × 10^−4^ (S · cm^−2^ · s^n^)	n_2_	R_ct_ (Ω· cm^2^)	χ^2^ (×10^−4^)
A: 5% NaCl	5.40	1.39	0.76	10,978	1.14	0.79	28,586	1.7
B: 10% NaCl	4.34	1.03	0.73	13,299	0.94	0.71	22,864	3.5
C: 5% NaNO_3_	6.63	1.23	0.90	33,576	1.50	0.94	82,576	3.1
D: 10% NaNO_3_	8.41	0.94	0.88	24,793	0.72	0.89	63,774	2.8
E: 5% NaCl + 5% NaNO_3_	7.17	1.32	0.81	17,474	2.28	0.75	25,472	4.6
F: 10% NaCl + 10% NaNO_3_	7.14	1.15	0.69	9,551	1.27	0.67	15,106	4.2

Cross-correlation analysis of electrochemical impedance spectroscopy parameters reveals profound insights into corrosion mechanisms. For instance, electrolyte F (10% NaCl + 10% NaNO_3_) exhibits low solution resistance, attributed to its highest overall ionic strength and conductivity. Notably, this electrolyte also exhibits the lowest charge transfer resistance. This correlation has a mechanistic basis: high chloride ion concentration enhances bulk solution conductivity while actively promoting interfacial corrosion. Abundant Cl^−^ ions efficiently adsorb and penetrate the passivation film, reducing its R_f_ and thereby catalyzing charge transfer reactions on the metal surface, leading to a significant decrease in R_ct_. This process is further corroborated by the non-ideal capacitive behavior exhibited at the interface in electrolyte F;the lowest CPE index (n_2_ = 0.67) indicates that localized corrosion leads to highly non-uniform surface morphology. In contrast, the high R_s values and exceptionally elevated R_ct_ values observed in NaNO_3_-dominated electrolytes (C and D) highlight the dual role of NO_3_^−^ ions. These ions simultaneously degrade the conductive environment while acting as inhibitory anions that stabilize the passivation film and suppress charge transfer reactions.

The CPE exponents n_1_ and n_2_ serve as critical metrics for assessing the morphology and homogeneity of the passive film. Deviations of these values from 1.0 indicate non-ideal capacitive behavior, stemming from non-uniform current and potential distributions across the electrode surface. The n_1_ parameter, associated with the outer passive film, is significantly influenced by surface roughness and porosity. The notably lower n_1_ values observed in chloride-containing electrolytes (e.g., 0.69 in fluoride solutions), compared to those in nitrate solutions (0.88–0.90 for C and D), suggest that Cl^−^ ions promote the formation of a more disordered and heterogeneous film-solution interface. This aligns with the mechanism of Cl^−^-induced localized etching and micropore formation. The n_2_ parameter, linked to the inner interface and charge transfer processes, reflects chemical and compositional heterogeneity at the substrate/film interface. Extremely low n_2_ values (down to 0.67 in electrolyte F) indicate a highly non-uniform distribution of electrochemical activity. This can be attributed to compositional non-uniformity within the passive film—the coexistence of regions rich in protective chromium oxide and less stable iron oxide—as well as the preferential adsorption of aggressive anions at specific active sites. Higher n_2_ values in nitrate solutions (approaching 1) suggest that NO_3_^−^ ions promote the formation of a more uniform and protective passive film, leading to a more ideal interfacial capacitance. The systematic differences between n_1_ and n_2_ highlight that these two electrochemical polarity coefficients correspond to distinct physical phenomena: outer interface geometry and inner interface chemical characteristics, respectively. The fitting results show that R_ct_ is the highest in 5% NaNO_3_ solution, indicating that the passive film structure is dense and has good protective performance. In 10% NaCl + 10% NaNO_3_ electrolyte, the high concentration of ions compresses the double-layer thickness, leading to a decrease in resistance. The Cl^−^ in the solution adsorbs on the surface of the passive film, reducing the activation energy barrier, resulting in a loose and porous passive film with poor protection, and thus the smallest R_ct_.

The observed passivation and failure behaviors in different electrolytes can be theoretically elucidated using established electrochemical models. In NaCl solutions, the point defect model (PDM) provides a mechanistic explanation for the destabilization of the passive film.^28^^,^^29^^,^^30^ According to this model, Cl^−^ ions promote the generation of metal vacancies at the metal/film interface, which can migrate and aggregate to form voids or defects, ultimately leading to film breakdown and localized dissolution. This aligns with our experimental results, which show increased corrosion depth and surface roughening in NaCl-rich environments. Conversely, in NaNO_3_ electrolytes, the enhanced passivation behavior can be qualitatively described by the Cabrera-Mott model,^31^ which explains the formation of thin oxide films via field-assisted migration of metal cations and oxygen anions. NO_3_^−^ can promote the rapid formation of a compact and protective passive film, which is consistent with the observed decrease in dissolution rate and smoother surface in NO_3_^−^ rich systems.

The electrochemical test results exhibit good agreement, as evidenced by the data obtained from the potentiodynamic polarization curves (passivation region range, passivation current density) and EIS (Electrochemical Impedance Spectroscopy) measurements. Specifically, 440C stainless steel demonstrates the widest passivation region, the lowest passivation current density, and the highest R_ct_ value in 5% NaNO_3_ solution, indicating significant capacitive behavior. Conversely, 440C stainless steel displays the smallest passivation region, the highest passivation current density, and the lowest R_ct_ value in 10% NaCl + 10% NaNO_3_ solution, suggesting inferior corrosion resistance. The EIS results corroborate the polarization curve findings, thereby validating the accuracy of the experimental outcomes.

Beyond the comparison of resistance values, the distribution of time constants offers deeper insights into the kinetics of interfacial processes. The time constants for the outer layer (τ_1_, associated with the passive film/electrolyte interface) and the inner charge transfer process (τ_2_, associated with the metal/passive film interface) were calculated through electrochemical impedance spectroscopy fitting parameters, as presented in Table 4.

**Table 4:** Time constant distribution in charge transfer processes

Electrolyte	τ_1_(s)	τ_2_(s)	τ_2_/τ_1_
A: 5% NaCl	0.20	0.55	2.8
B: 10% NaCl	0.12	0.30	2.5
C: 5% NaNO_3_	4.71	12.10	2.6
D: 10% NaNO_3_	1.81	5.22	2.9
E: 5% NaCl + 5% NaNO_3_	0.38	0.96	2.5
F: 10% NaCl + 10% NaNO_3_	0.11	0.22	2.0

The data in the table reveal that the τ_2_/τ_1_ ratio for all systems exceeds 2.0, confirming sufficient separation of the two time constants and validating the reliability of the proposed equivalent circuit model in decoupling the two interfacial processes. The pure NaNO_3_ solution (C and D) exhibits the highest absolute τ values, indicating that the kinetics at both interfaces are more sluggish and inhibited, which is consistent with the characteristics of the passive film formed in the nitrate solution. Conversely, the presence of chloride ions (solutions A, B, E, and F) significantly reduces the values of τ_1_ and τ_2_. This suggests an acceleration of the electrochemical reactions at both interfaces. Chloride ions adsorb on the passive film surface, compromising its stability and promoting ion transport through the film layer (shortening τ_1_). Subsequently, they also accelerate the charge transfer reaction at the underlying metal surface (shortening τ_2_), thereby promoting localized corrosion. Furthermore, the CPE behavior (*n* < 1) in Table 3 further corroborates the non-ideal characteristics of these interfaces. The lower *n* values observed in chloride-containing environments (particularly in the charge transfer process, such as n_2_ ≈ 0.67–0.71 in solutions B and F) indicate increased surface heterogeneity and disorder. This is likely a result of the localized breakdown of the passive layer and the formation of active corrosion sites, leading to a much stronger coupling between the dissolution of the passive layer and the charge transfer phenomenon compared to the uniform protective passive layer formed in the nitrate environment.

**Figure 8:**
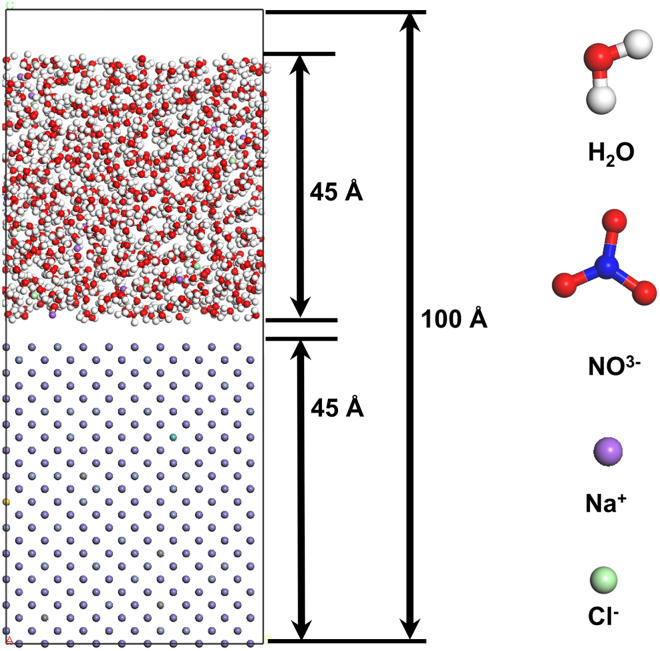
Schematic close-up view of simulated 440C stainless steel and 5% NaCl electrolyte (left side); ionic modeling of elements contained in the simulation (right side)

To investigate the influence of different electrolytes on the electrochemical corrosion behavior of 440C stainless steel at a microscopic level, MD models were established for 440C stainless steel in six electrolytes using Material Studio software, as illustrated in Figure 8.

The L-J potential parameters used in this study are summarized in Table 5. To improve computational efficiency, the Shake algorithm was employed to constrain bond lengths, and the Verlet algorithm was used for numerical integration of particle trajectories.^32^

**Table 5:** Potential function

Atom pairs	*ε*/*eV*	*σ*/*Å*
Fe-Fe	0.055	4.54
Fe-Cr	0.0287	3.7046
Fe-N	0.1311	4.0969
Fe-O	0.0923	3.791
Fe-H	0.0555	3.2747
Fe-Na	0.0406	3.6801
Fe-Cl	0.0353	4.2331
Cr-Cr	0.015	3.023
Cr-N	0.0685	3.3431
Cr-O	0.0482	3.0934
Cr-H	0.029	2.6721
Cr-Na	0.0212	3.0029
Cr-Cl	0.0185	3.4542
N-N	0.3125	3.697
N-O	0.2201	3.421
N-H	0.1323	2.955
N-Na	0.0968	3.3209
N-Cl	0.0842	3.82
O-O	0.155	3.1655
O-H	0.0932	2.7344
O-Na	0.0682	3.0729
O-Cl	0.0593	3.5347
H-H	0.056	2.362
H-Na	0.041	2.6544
H-Cl	0.0357	3.0533
Na-Na	0.03	2.983
Na-Cl	0.0261	3.4313
Cl-Cl	0.0227	3.947

**Figure 9:**
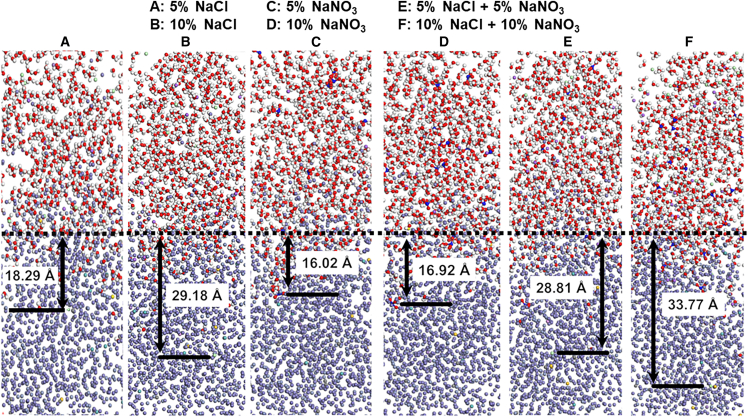
Simulation results and corrosion distances of electrochemical corrosion in different electrolyte environments (A) shows 5% NaCl solution, (B) shows 10% NaCl, (C) shows 5% NaNO_3_, (D) shows 10% NaNO_3_, (E) shows 5% NaCl + 5% NaNO_3_, and (F) shows 10% NaCl + 10% NaNO_3_. The distances between the different regions are indicated in Angstroms.

As shown in Figure 9, the corrosion process in different electrolytes was evaluated by tracking the intrusion of Cl^−^ and NO_3_^−^ ions into the material and the escape of Fe^3+^ and Cr^3+^ ions from the surface.

Simulation results indicate that the corrosion distance in the composite electrolyte F is greater than that in other electrolytes. When comparing the corrosion distances in single-component electrolytes, the NaCl solution exhibits a greater corrosion depth than the NaNO_3_ electrolyte. This can be attributed to the oxidative effect of NO_3_^−^ ions, which oxidize Fe^2+^ to Fe^3+^ during electrolysis. The increased Fe^3+^ content facilitates the formation of a Fe_3_O_4_ passive film, thereby impeding the progression of electrochemical corrosion.^33^ The relationship between corrosion rates and corrosion distances remains consistent in different electrolyte environments. By comparing simulation outcomes under varying initial conditions, convergence was ensured. Furthermore, a reduced time step was utilized throughout the simulation to mitigate error propagation. In order to minimize the impact of equipment factors, we performed three calculations for each set of parameters. The corrosion rates and distances in different electrolytes are presented in Table 6.

**Table 6:** Corrosion rates and distances in different electrolyte environments in simulations

Electrolyte	Corrosion distances (Å)	Corrosion rates (g/(min·mm^2^))
A: 5% NaCl	18.29 ± 1.65	0.98 ± 0.17
B: 10% NaCl	29.18 ± 1.52	1.91 ± 0.16
C: 5% NaNO_3_	16.02 ± 1.27	0.71 ± 0.09
D: 10% NaNO_3_	16.92 ± 1.34	0.83 ± 0.14
E: 5% NaCl + 5% NaNO_3_	28.81 ± 1.98	1.83 ± 0.12
F: 10% NaCl + 10% NaNO_3_	33.77 ± 1.85	2.09 ± 0.11

**Figure 10:**
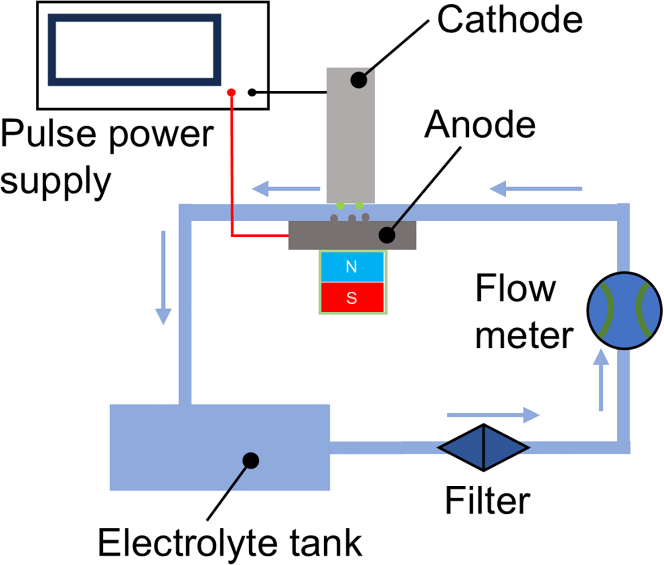
Schematic diagram of electrochemical corrosion

To investigate the electrochemical dissolution behavior of 440C stainless steel in a neutral electrolyte and examine the influence of an external magnetic field on the efficiency and surface quality of electrochemical corrosion, an electrochemical corrosion apparatus was constructed, as shown in Figure 10. An NdFeB magnet was placed beneath the anode, 304 stainless steel was used as the cathode, and a high-frequency pulse power supply provided the electrical input. The corrosion parameters are listed in Table 7.

**Table 7:** Electrochemical corrosion parameters

Parameter	Value
Pulse power frequency	5,000 Hz
Duty cycle	50%
Peak voltage	20 V
Corrosion gap	0.2 mm
Electrolyte flow rate	2 m s^−1^
Temperature	25 ± 1°
Corrosion time	3 min

A sample of 440C stainless steel sized at 20 mm × 20 mm × 2 mm was selected, and six types of neutral solutions were prepared as the electrolytes for the electrochemical corrosion experiments, with the experimental arrangement detailed in Table 8. To ensure data reliability, triplicate experiments were conducted. After the experiments, the samples were placed in deionized water for ultrasonic cleaning and drying. The mass of each sample before and after the experiments was measured using a precision balance, and the MRR under different electrolyte and magnetic field conditions was calculated. The surface morphology of 440C stainless steel was examined using a VT6200 large stroke confocal microscope (Chotest Technology Inc [chotest]), with surface roughness selected as the evaluation criterion. Scanning electron microscopy (SEM) was used to investigate the micro-morphology of the corrosion zones under various corrosion conditions, in order to study the effects of the electrolyte composition and applied magnetic field on the electrochemical corrosion surface.

**Table 8:** Experimental arrangement and results without magnetic field

Sample	Electrolyte	MRR/g·min^−1^	Ra/μm
Substrate	/	/	2.439 ± 0.138
a	A: 5% NaCl	0.027 ± 0.007	2.269 ± 0.106
b	B: 10% NaCl	0.036 ± 0.005	2.790 ± 0.189
c	C: 5% NaNO_3_	0.014 ± 0.002	1.454 ± 0.136
d	D: 10% NaNO_3_	0.019 ± 0.003	2.086 ± 0.117
e	E: 5% NaCl + 5% NaNO_3_	0.032 ± 0.005	2.380 ± 0.095
f	F: 10% NaCl + 10% NaNO_3_	0.042 ± 0.006	3.236 ± 0.168

As shown in Table 8, under the same electrolyte conditions, a higher electrolyte concentration leads to greater conductivity and faster electrochemical reaction rates within the same time frame, thereby resulting in a relatively larger MRR. Compared to NO_3_^−^ ions, Cl^−^ ions possess a smaller ionic radius and higher permeability, making it easier for them to penetrate the surface passivation films and place the material in an activated dissolution state. Consequently, 440C stainless steel exhibits a higher MRR for in NaCl electrolyte. In contrast, due to its strong oxidative nature, NO_3_^−^ tends to promote the formation of a passivation films on the material surface, leading to a lower MRR in NaNO_3_ electrolyte. For mixed electrolytes with equal concentrations of NaCl and NaNO_3_, electrochemical corrosion reflects the combined effects of Cl^−^ induced activation and NO_3_^−^ induced passivation, resulting in an MRR value that falls between those observed in the single-component electrolytes.

The surface roughness of 440C stainless steel after electrochemical corrosion in electrolytes that do not contain 10% NaCl is lower than that of the original surface when no external magnetic field is applied. This is attributed to the easy penetration of Cl^−^ ions through the passive film on the material surface, where they combine with cations in the film to form soluble chlorides. This process leads to localized pitting corrosion and uneven material dissolution. Electrolytes with higher NaCl concentrations exhibit greater conductivity, accelerating electrochemical reactions and exacerbating surface unevenness, thereby increasing surface roughness. In contrast, NaNO_3_ is a passivating electrolyte that promotes the formation of a dense passive film on the 440C stainless steel surface. A higher voltage is required to disrupt this film. Under such conditions, surface protrusions are more easily dissolved, while the passive film undergoes cyclic processes of dissolution, regeneration, and re-dissolution, ultimately leading to a smoother surface with reduced roughness.

To investigate the effect of magnetic field on the electrochemical corrosion behavior of 440C stainless steel, an NdFeB magnet was placed beneath the anode. The MRR and surface roughness of the 440C stainless steel under the influence of the magnetic field were evaluated, as summarized in Table 9.

**Table 9:** Experimental arrangement and results with magnetic field (100 mT)

Sample	Electrolyte	MRR/g·min^−1^	Ra/μm
Substrate	/	/	2.439 ± 0.138
a-M	A: 5% NaCl	0.034 ± 0.006	1.817 ± 0.161
b-M	B: 10% NaCl	0.045 ± 0.002	2.026 ± 0.192
c-M	C: 5% NaNO_3_	0.021 ± 0.005	0.824 ± 0.064
d-M	D: 10% NaNO_3_	0.024 ± 0.004	1.702 ± 0.175
e-M	E: 5% NaCl + 5% NaNO_3_	0.040 ± 0.004	1.880 ± 0.075
f-M	F: 10% NaCl + 10% NaNO_3_	0.054 ± 0.007	2.208 ± 0.103

## Discussion

Current density is a significant evaluation criterion in the electrochemical corrosion,^34^ and the influence of a magnetic field on current density is as follows^35^:

(Equation 5) J=σE+σu×B

where *J* represents current density, *σ* denotes electrolyte conductivity, *E* stands for electric field strength, *u* represents velocity field, and *B* is the magnetic induction intensity.

The introduction of a magnetic field enhances the current density and electrochemical reaction rate within the electrochemical reaction zone. Additionally, charged particles in the electrolyte are influenced by both the electric field force and Lorentz force in the corrosion zone, altering their motion—a phenomenon known as magnetohydrodynamics. This effect induces disturbances in the electrolyte, promoting electrolyte renewal and the timely removal of corrosion products. Consequently, the negative impact on conductivity and current density in the corrosion zone is reduced. Therefore, under the influence of an external magnetic field, 440C stainless steel exhibits varying degrees of increased MRR in the electrolytes studied.

**Figure 11:**
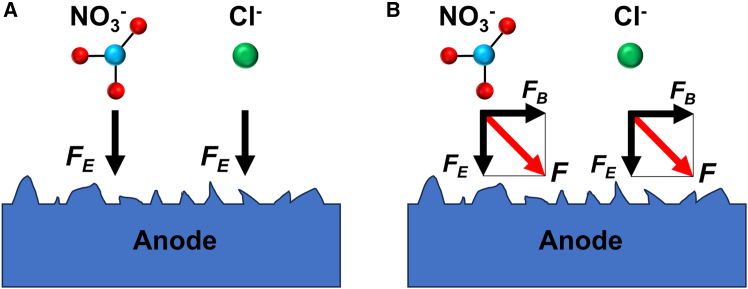
Modeling of ion migration in electrolytes (A) Electric field effect; (B) joint action of electric and magnetic fields.

In the absence of a magnetic field, electrolyte ions in the corrosion zone are driven solely by the electric field force (*F*_*E*_), moving directionally from the cathode to the anode, perpendicular to the anode surface. This results in synchronized erosion of the anode surface, including both protrusions and pits, as illustrated in Figure 11A. When an external magnetic field is applied, the ions in the solution are subjected to the combined action of the electric field force (*F*_*E*_) and the magnetic Lorentz force (*F*_*B*_), altering their migration direction. Under the resultant force, the ions approach the anode surface at an inclined angle, which facilitates preferential dissolution of the protruding structures on the surface.^36^ Furthermore, the altered ion migration induces disturbances in the electrolyte, promoting the removal of corrosion by-products and ensuring a more uniform distribution of electrolyte conductivity and current density in the corrosion zone. As a result, the surface roughness is reduced.

**Figure 12:**
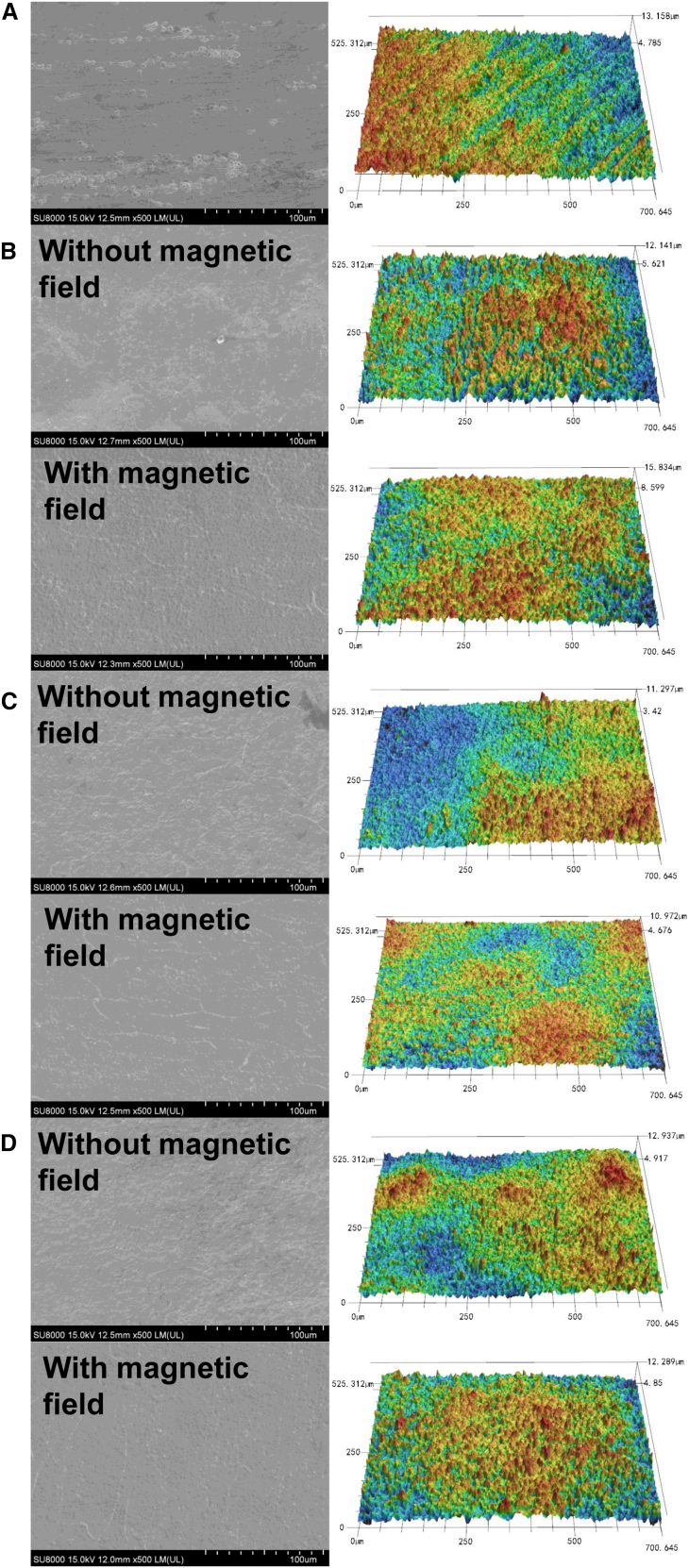
Surface morphology and 3D profile of 440C stainless steel (A) Substrate; (B) under 10% NaCl electrolyte conditions; (C) under 10% NaNO_3_ electrolyte conditions; (D) under 5% NaCl + 5% NaNO_3_ electrolyte conditions.

SEM images and 3D profiles of the 440C stainless steel substrate after electrochemical corrosion under different electrolyte and magnetic field conditions are presented in Figure 12. Figure 12A reveals pronounced mechanical corrosion marks on the surface of the untreated 440C stainless steel substrate, characterized by distinct protrusions and cavities with significant surface undulations. After electrochemical corrosion, Figures 12B–12D show that these mechanical marks are no longer visible. Figure 12B compares the surface morphology before and after magnetic field application in a 10% NaCl electrolyte. Without the magnetic field, the strong penetration capability of Cl^−^ ions results in uneven surface erosion. This is evidenced by noticeable protrusions in the SEM image and a wide color variation in the 3D surface profile. When the magnetic field is applied, the surface becomes relatively smoother, with fewer prominent protrusions and cavities, and a more uniform color distribution in the 3D profile. Similar trends are observed in the 10% NaNO_3_ and 5% NaCl + 5% NaNO_3_ electrolytes, as shown in Figures 12C and 12D. These results indicate that the application of a magnetic field reduces the surface roughness of 440C stainless steel during electrochemical corrosion.

### Conclusions

This study systematically investigates the electrochemical corrosion behavior of 440C stainless steel in various electrolytes through electrochemical measurements, MD simulations, and corrosion experiments. The key findings are summarized as follows.

(1) In a 5% NaNO_3_ electrolyte, 440C stainless steel exhibits the widest passivation range (1,180 mV) and the highest charge transfer resistance (R_ct_ = 82,576 Ω cm^2^), indicating the formation of the most stable and dense passive film.
(2) Conversely, a 10% NaCl + 10% NaNO_3_ electrolyte demonstrates the narrowest passivation range (196 mV) and the lowest Rct, suggesting reduced passivation stability and corrosion resistance.
(3) MD simulations reveal that Cl^−^ ions significantly enhance electrolyte permeability. The 10% NaCl + 10% NaNO_3_ electrolyte leads to the greatest corrosion depth (33.77 Å) and the highest corrosion rate (2.09 g/(min·mm^2^), while 5% NaNO_3_ shows the smallest penetration depth (16.02 Å) and the lowest rate (0.71 g/(min·mm^2^).
(4) Electrochemical corrosion experiments confirm that the mixed electrolyte (10% NaCl + 10% NaNO_3_) produces the highest MRR but also results in the highest surface roughness. In contrast, 5% NaNO_3_ provides a smoother surface but a lower MRR.
(5) The application of an external magnetic field enhances electrochemical reactions, increases MRR, and simultaneously reduces surface roughness, thereby optimizing corrosion efficiency and surface quality.
(6) The consistency between electrochemical results, MD simulations, and corrosion experiments validates the reliability of the MD method in simulating electrochemical corrosion mechanisms.

Overall, the selection of electrolyte composition and the application of a magnetic field should balance dissolution efficiency and surface integrity to optimize the electrochemical corrosion performance of 440C stainless steel.

### Limitations of the study

This study employs MD simulations based on classical force fields to investigate the fundamental transport behavior of electrolyte ions within a passive film. It is important to note that this work may not fully capture the complex charge transfer and chemical bonding interactions at the metal-oxide interface. The idealized atomic structure of the passive film may not fully represent the heterogeneous features present in experimental samples, such as grain boundaries and vacancies, potentially leading to deviations in the quantitative diffusion coefficients and interactions. However, this model can still provide qualitative trends relevant to the study of the corrosive behavior of electrolyte solutions on materials. Future research could consider employing more sophisticated models, such as incorporating more surface effects and the non-ideality of the solution, to enhance the accuracy of the simulation results. Moreover, we conducted an initial analysis of the influence of an external magnetic field but did not delve into the non-linear effects of magnetic field strength on the corrosion rate. Therefore, future work could further investigate the critical values and influencing mechanisms of magnetic field strength to better understand its role under various conditions. While this study elucidates the interaction mechanisms between ions and the passivation layer through experimental and MD analyses, it does not incorporate detailed thermodynamic or kinetic modeling of the passivation behavior. Future research will consider the application of kinetic models, such as the Cabrera-Mott theory or PDMs, and thermodynamic calculations to quantitatively analyze the growth, stability, and breakdown processes of the passivation film under different electrolyte conditions.

## Resource availability

### Lead contact

Further information and requests for resources should be directed to and will be fulfilled by the lead contact, Xinyu Wen (18865721932@163.com).

### Materials availability

This study did not generate new unique reagents.

### Data and code availability

• All data reported in this paper will be shared by the lead contact upon request.
• This paper does not report original code.
• Any additional information required to reanalyze the data reported in this paper is available from the lead contact upon request.

## Acknowledgments

The project is supported by the Tongling Major Science and Technology Program (202101JB003), Anhui Province Key Laboratory of Critical Friction Pair for Advanced Equipment (LCFP2406, 2024hxkt2024073), Major Project of Scientific Research in Higher Education Institutions of Anhui Province (2024AH040123), Anhui Province Scientific Research Programming Project (2024AH051984), and Scientific Research Initiation Grant Program for High-level Talents of 10.13039/501100020453West Anhui University (WGKQ2022014).

## Author contributions

X.W.: conceptualization, methodology, software, validation, data curation, writing – draft, writing – editing, and visualization. Y.C.: conceptualization, formal analysis, resources, supervision, project management, and funding acquisition. F.Z.: conceptualization, methodology, software, data curation, writing – editing, and visualization. X.L.: software, investigation, data curation, visualization, and funding acquisition. H.L.: investigation, data curation, visualization, and funding acquisition. W.J.: resources, supervision, and project management.

## Declaration of interests

The authors declare no competing interests.

## STAR★Methods

### Key resources table

**Table:** 

REAGENT or RESOURCE	SOURCE	IDENTIFIER
**Chemicals, peptides, and recombinant proteins**		

NaCl	Aladdin	MFCD00003477; CAS: 7647-14-5
NaNO_3_	Aladdin	MFCD00085307; CAS: 7631-99-4

**Software and algorithms**		

OriginPro2023	OriginLab	https://www.originlab.com/
MD software, Materials Studio 2020	Dassault Systèmes	https://www.3ds.com/zh-hans/products/biovia
ZView	Scribner	https://www.scribner.com/software/68-general-electrochemistr376-zview-for-windows/

### Method details

#### Pre-treatment method

A 440C stainless steel specimen (20 mm × 20 mm × 2 mm) was selected as the substrate. It was mechanically polished to remove the surface oxide layer, immersed in 30% HCl solution for 20 s to eliminate residual impurities and oxides, and finally ultrasonically cleaned in deionized water for 10 min.

#### Electrochemical testing method

The dissolution behavior of 440C stainless steel in various electrolytes was investigated using a three-electrode system with an electrochemical workstation (CHI660E CH Instruments, China). A platinum electrode served as the counter electrode, a saturated calomel electrode (SCE) as the reference electrode, and 440C stainless steel as the working electrode.

#### MD modeling method

The 440C stainless steel model was constructed based on the elemental composition obtained from EDS Mapping scans, with a Fe (1 1 1) crystal supercell randomly doped via a script to reflect the actual elemental composition of 440C stainless steel. For simulating various electrolyte environments, water molecule models were built using the Simple Point Charge Extended (SPC/E) and Transferable Intermolecular Potential 4 Points (TIP4P) water models,^37^ incorporating the required electrolyte ions such as NO_3_^−^, Na^+^, Cl^−^, along with 1000 water molecules and 50 different ions. The final idealized atomic model of 440C stainless steel contained 3000 atoms.

The classical canonical ensemble (NVT) was employed, with temperature regulation implemented via the Nose-Hoover thermostat to maintain system energy equilibrium and ensure a constant temperature of 298 K throughout the simulations.^38^ The Particle-Particle-Particle-Mesh (PPPM) algorithm was utilized to enhance the accuracy of long-range electrostatic interactions among charged atoms.^39^ Interatomic interactions were modeled using the Lennard-Jones potential energy function,^40^^,^^41^ as described by Equation:

$$V\left(r\right)=4\varepsilon \left[{\left(\frac{\sigma }{r}\right)}^{12}-{\left(\frac{\sigma }{r}\right)}^{6}\right]$$

Here, *ε* represents the basic energy parameter of the particles, *σ* is the distance parameter when the interaction potential between particles is 0, and *r* is the distance between particles.^42^

#### MD simulation boundary condition

Under identical conditions of temperature, time, and applied voltage, the corrosion behavior of 440C stainless steel was investigated in various electrolyte solutions. The simulation was conducted at 25 °C, for a duration of 200 ps, and with an applied voltage at 10 V.

### Quantification and statistical analysis

#### Definition of corrosion distance and calculation method for corrosion rate

The corrosion distance at a simulation time of 200 pico seconds was defined as the distance traveled by anions from the equilibrium layer. By using MD simulation software, the number of atoms that intruded into or escape from the equilibrium layer per unit time was extracted to calculate the electrochemical corrosion rates in each electrolyte systems, as shown in Equation.^43^

$$V=\frac{\Delta m}{AT}$$

Where, *V* represents the electrochemical corrosion rate, Δ*m* denotes the absolute change in mass of the equilibrium layer, *A* is the contact area of the equilibrium layer, and *T* represents the time of the corrosion process.
